# Education for public health in Europe and its global outreach

**DOI:** 10.3402/gha.v7.23570

**Published:** 2014-02-13

**Authors:** Vesna Bjegovic-Mikanovic, Aleksandra Jovic-Vranes, Katarzyna Czabanowska, Robert Otok

**Affiliations:** 1 University of Belgrade, Faculty of Medicine, Centre School of Public Health and Management, Belgrade, Serbia; 2 Department of International Health, CAPHRI School for Public Health and Primary Care, Faculty of Health, Medicine and Life Sciences, Maastricht University, Maastricht, The Netherlands; 3 Association of Schools of Public Health in the European Region (ASPHER), Brussels, Belgium

**Keywords:** education, public health, global health, competences, continuing professional development, networking

## Abstract

**Introduction:**

At the present time, higher education institutions dealing with education for public health in Europe and beyond are faced with a complex and comprehensive task of responding to global health challenges.

**Review:**

Literature reviews in public health and global health and exploration of internet presentations of regional and global organisations dealing with education for public health were the main methods employed in the work presented in this paper. Higher academic institutions are searching for appropriate strategies in competences-based education, which will increase the global attractiveness of their academic programmes and courses for continuous professional development. Academic professionals are taking advantage of blended learning and new web technologies. In Europe and beyond they are opening up debates about the scope of public health and global health. Nevertheless, global health is bringing revitalisation of public health education, which is recognised as one of the core components by many other academic institutions involved in global health work. More than ever, higher academic institutions for public health are recognising the importance of institutional partnerships with various organisations and efficient modes of cooperation in regional and global networks. Networking in a global setting is bringing new opportunities, but also opening debates about global harmonisation of competence-based education to achieve functional knowledge, increase mobility of public health professionals, better employability and affordable performance.

**Conclusions:**

As public health opportunities and threats are increasingly global, higher education institutions in Europe and in other regions have to look beyond national boundaries and participate in networks for education, research and practice.

Nowadays, from various angles, global health is challenging education for public health. Schools, departments, and institutes of public health, as members of the European Higher Education Area (EHEA), are to train their students and public health professionals to be able to develop, organise, manage, evaluate, and adjust interventions aiming at the promotion of health and at the reduction of present and forecasted public health challenges. In most if not all countries, the role of education and capacity building for global health is recognised by many stakeholders in public health, responding to global efforts to reduce the burden of disease, strengthen the health systems, and disseminate scientific innovation (1, 2). This is a complex and comprehensive task for two reasons:

1. The health sciences – providing the framework for education and training for public health – are an ensemble of individual disciplines stemming from two scientific paradigms, the medical and natural sciences on the one hand and the social sciences on the other. However, they focus on a common subject, the analysis of and evidence-based intervention in health and disease processes, determined by population, environment and social system (including the health system) (3);
2. The target group of public health students and public health professionals is a very heterogeneous one and they must be able to handle population health problems as well as public health systems and other man-made systems with health impact in a national and global context, in order to meet the challenges of increasing healthy life years and reduce health inequalities. To that end, they have to communicate with different actors, joined in multidisciplinary teams of researchers, institutional decision makers, and representatives of government, civil society and the private sector.

## Global background

Cooperation between academic institutions and civil society becomes increasingly important in capacity building of public health professionals, particularly in addressing the global health agenda, with a lot of examples of good practice and lessons learnt (4). Debates about the scope of education for global health, the complementarities in basic definitions of global health and public health (5, 6) and ethical considerations of partnerships involved in education (7), are taking advantage from community engagement and civil society organisations. The goals of public health training in the 21st century, brilliantly defined as follows, are also applicable to global health training (8): stimulate curiosity to examine evidence and to provide appropriate health interventions; produce graduates who are taking initiatives, accept risk and responsibility for failures; who are capable to make connections, to understand root causes of health and illness and refocus upstream and downstream to reach gain in population health, with self-confident behaviour when they advocate for health and human rights.

Virtual communities – new educational technologies and virtual universities/classrooms, based on digital media – are supporting equal opportunities and equal gain in educational outcomes for many students and public health professionals worldwide (9). A road map for the marketing and long-term sustainability of academic programmes has often been seen in the enhancement through innovative educational technologies. Institutions of higher education across the world are responding to the challenges and opportunities presented by the availability of new Web 2.0 applications. Taking advantage of new technologies, there is a lot of experience in establishing regional and global networks dealing with the challenges of global health, besides the well-established system of international organisations within the United Nations and beyond. However, the global community is still struggling to harmonise efforts, witnessing slow progress in reaching equal benefits for all countries from such partnerships (10). A recent example is the International Health Partnerships (IHP+) (11).

Both education and research are core composite parts in the development of globalisation, while cross-border mobility of higher education is stimulating the higher education institutions to become global actors. This process is characterised by increasing trends of students’ and teachers’ mobility and growing diversity of educational programmes in terms of institutional autonomy, funding, and quality assurance (12). University partnerships are the most developed form of cooperation. Recent OECD statistics pointed out that the number of international and foreign students increased by 90% in the last years, to be around 2.5 million globally, while Australia, the United Kingdom, Germany and France remain among the major hosting countries, and the United States dropped from the first to the 29th rank in relative figures (per total enrolment). In absolute terms, the United States remains the most attractive country for foreign students (13). According to the latest survey among European Schools and Departments of Public Health, members of the Association of Schools of Public Health in the European Region (ASPHER), there are international students make up 20.5% out of the total enrolment (14). In general, higher education and training is seen as one of the pillars of competitiveness in the global economy, still struggling with crisis and recovery (15). Therefore, many higher education institutions are increasing competitive advantage by employing marketing strategies for better visibility of their programmes, supplied to the global market. The most frequently applied strategies are based on mutual understanding, excellence and competition for talents, revenue generation and capacity building focussed on economic considerations (12).

New trends in higher education and the struggle for sustainable development beyond 2015, with special reference to the Millennium Development Goals, make global health an emerging topic of highest relevance for academic institutions, both in medicine and public health. The increasing demand for education in global health becomes apparent in students associations’ demands to integrate global health as a topic of academic curricula. An example is the voice of the International Federation of Medical Students Associations, representing more than 1 million students around the globe and having a standing committee on public health with many initiatives in global health (16). The number of academic programmes in global health is progressively increasing and many authors are pointing to the need of equal partnerships between high and low/middle developed countries, as well as measuring their impact on leadership development, strengthening of health systems and scientific capacities (2). The commonality of educational programmes for global health is their mission to contribute through education and training to decrease the global burden of diseases, increase life expectancy and support equal access to health services. Nevertheless, just as unequal distribution of economic wealth and huge disparities in health are obvious throughout the world, the same is true for the distribution of academic programmes for education and training in global health, being much more frequently organised by institutions in high income countries (17). Additional misbalance and failure are related to lack of involvement of recipient countries in setting priority topics for training in global health. Furthermore there is improper selection of students who are not involved, nor plan to be involved, in applying what they learned in real life. Finally low/middle income countries often do not have the resources to organise high quality training. In order to avoid misbalance, reduce failure and provide gain for all participants involved in global health education, there are several initiatives to develop ethical guidelines for global health training related to sending and hosting institutions, trainees, and sponsors (18). A recent evaluation of curricula related to the ethical dimension of training programmes in global health showed that only 31% of those who were involved in the delivery of education for global health had prior ethical training (19).

## The European framework for public health education and training in global health

Looking at education for public health in Europe and its global outreach, there are many vivid initiatives as health is recognised as a core determinant of social cohesion and the major factor of peace building, investment and overall sustainable development. The Bologna process and the Lisbon Strategy in Europe are often cited as the first international documents for higher education involving more than 40 countries, and followed by other regions in the world (20). Today a new movement for globalisation of education for health is expressed in many academic networks for public health in Europe, flourishing during the last decade and scaling up research, education and practise (21, 22). This renewed approach is also in line with WHO Regional Office's new European strategy and framework for health, Health 2020, where investing in capacity for public health, change, innovation and leadership constitute key action principles. Moreover, the European Union's (EU) ‘Europe 2020’ strategy puts knowledge and innovation at the heart of the EU's blueprint for competitiveness. Finally, a big first step has been taken with the development of the WHO European Action Plan for Strengthening Public Health Capacities and Services (23). ASPHER has already summarised the challenge in the early 90s, creating the slogan ‘Training for Public Health Practice and Research’.

As part of the initiatives for social cohesion, addressing disparities in health and quality of life between and within countries in Europe, investment in strengthening of public health functions and development of public health professional capacity is of key importance. The European vision of a poverty-free, educated and healthy society, united in economic and social development has a solid base in the Bologna process. Following the Bologna process, through Berlin (2003), Bergen (2005), London (2007) and Leuven/Louvain-la-Neuve Communiqués (2009), the European Ministries of Education have adopted the Budapest–Vienna Declaration on the EHEA in March 2010. Higher education priorities in the decades to come are focussing on the social dimension of education (equitable access and completion), lifelong learning, student-centred learning and highly motivated teachers, mobility, employability, and international openness (24). In the Bucharest Communiqué (2012), besides agreeing on the same priorities for 2010–2020, the role of higher education is set up to increase Europe's capacity in dealing with the economic crisis and contributing to growth and jobs (25). One of set priorities is to evaluate the implementation of the ‘EHEA in a Global Setting’ Strategy up to 2015 with the basic aim to provide guidelines for a further global development of EHEA (26). The guiding strategic principles are ‘European heritage and values, stakeholder participation and geographical scope’, while key policy areas reflect steps in the implementation process for internationalisation of EHEA. These are also strong prerequisites in Europe for involvement in the education for global health.

On the same line are all actions of the EU in the field of higher education, starting from the Lisbon Strategy and ‘The role of education in a fully-functioning knowledge triangle’ (27), while later these actions are supported by fully opening towards global health (The EU Role in Global Health) (28). The ‘Strategic framework for European cooperation in education and training’ (‘ET 2020’) (29) has also established respective strategic objectives and their achievement is supported by performance standards in the field of education (‘European benchmarks’) (30). Out of five benchmarks, two are certainly relevant for public health education and training in the context of global health:

1. Adult participation in lifelong learning: By 2020, an average of at least 15% of adults (25–64) should participate in lifelong learning.
2. Tertiary level attainment: By 2020, the share of 30–34 year olds with tertiary educational attainment should be at least 40%.

## The importance of competence-based education in the European approach to global health

Since the 90s, in Europe (31, 32), training programmes are increasingly structured around exit competences, in terms of what the graduate should be able to do in order to achieve certain performance standards expected by future employers. Public health competences may be defined as a complex set of measurable behaviours made up of knowledge, skills and attitudes that can be shown to predict and measure effective performance (33). WHO defines competence even more precisely as the combination of technical knowledge, skills and behaviours (34). Competence-based education (CBE) is organised around competences, or predefined abilities, as outcomes of the curriculum. ‘Competences’ have become the units of medical and public health educational planning (35).

In order to assure that the Schools of Public Health adequately address the skill needs of the employment market, close partnerships are needed between employers and educators, both of which are essential partners of a ‘knowledge triangle’ based on the interaction of education, research and innovation (36). Employers consider tacit knowledge, generic skills and work-based attitudes as more important than academic or technical knowledge, which they take for granted when employing graduates holding certain degrees in public health (37). In fact, the most important skill that Europe's public health professionals will need in order to adapt to the demands of the future is the ability to be lifelong learners irrespective of the discipline.

There is growing consensus – e.g. in the US, Canada, and other countries, and today also in Europe – on the key competence areas in academic public health curricula. All of them are relevant for global health reflecting the cross-disciplinarity of the sciences involved in public health (31). The following six main domains of public health competences in Europe are developed by the ASPHER community: methods in public health (epidemiology and biostatistics); population health and its social determinants, environmental health sciences; health policy, management of health services and health economics; health promotion (health education, health protection and disease prevention); and ethics. The set of competences in each group is unlimited, defined by new challenges in the future. Their application depends on adequate public health problem identification, best achieved through community and situation analysis, selection of targets and identification of target groups, selection of intervention, implementation, follow-up and evaluation.

European competences are comparable to those developed by other organisations such as the Association of Schools of Public Health (ASPH) in the US (38). There are similar projects which aim at the development of more specific lists of competences in Europe such as the Core Competences Framework for Health Promotion (39), Core Competences for Public Health Epidemiologists (40), or competences in the area of Public Health Leadership (41).

The development of public health competence sets logically leads to formalised evaluation and accreditation of training programs. Therefore, ASPHER has taken the initiative, together with partners – EUPHA, the European Public Health Alliance (EPHA), the European Health Management Association (EHMA), and Euro-HealthNet – and in consultation with WHO Europe and the European Commission, to establish a European Agency for Public Health Education Accreditation (APHEA) for the accreditation of public health educational programmes and schools of public health (42).

Following the demand of students, specific global health competences are developed and evaluated throughout the world, particularly for graduates who attend medical education (43–45). In fact, competences related to public health are listed as: global burden of disease, health implications of travel, migration and displacement, social and economic determinants of health, population, resources and environment, globalisation of health and healthcare, healthcare in low-resource settings, human rights in global health, cultural diversity and health (46, 47). In contrast to higher education in medicine, so far only in the United States are competences defined within public health specifically as a Global Health Competency Model (48). In Europe, such a model is not yet developed. However, starting from the statement that ‘global health is public health’, schools of public health are well-equipped to deal with global health challenges and be responsible for promoting education, research and capacity building in global health (Table 1).

**Table 1:** Selected list of competences’ domains in global health for medical and public health students

Medical students		Public health students
US, Canada: Global Health Education Consortium^a^	UK: the Global Health Learning Outcomes Working Group^b^	ASPH: Global Health Competency Model^c^
Global burden of disease	Global burden of disease	Capacity strengthening
Health implications of travel, migration and displacement	Socioeconomic and environmental determinants of health	Collaborating and partnering
Social and economic determinants of health	Health systems	Ethical reasoning and professional practice
Population, resources, and environment	Global health governance	Health equity and social justice
Globalisation of health and healthcare	Human rights and ethics	Program management
Healthcare in low-resource settings	Cultural diversity and health	Socio-cultural and political Awareness
Human rights in global health		Strategic analysis

a Global Health Education Consortium (47)

b Johnson et al. (46)

c ASPH (48).

This is also stated in the Declaration of the Third Global Summit of Schools of Public Health (49). The Global Summit was initiated by the European Academic Global Health Alliance (EAGHA), which is supported and hosted by ASPHER in Europe. In 2012, EAGHA released a Global Health Education Declaration, with the initial target audience being medical schools offering primary qualifications for physicians (50).

## Schools and Departments of Public Health (SDPH) in Europe and their global outreach

The general task profile of schools of public health in Europe can be described as follows (51):

1. Training for research and services
2. Monitoring population health
3. Community oriented interventions
4. Liaising with Public Health Associations
5. Consulting with decision makers

The European SDPH seems to be willing to take up global health as a high level teaching subject. In the recent survey by ASPHER of SDPH in Europe,^1^ it emerged however that capacity is very limited: members of ASPHER are usually public institutions, most based at universities with a median of 20 Full-Time-Equivalent (FTE) lecturing staff, 86.4% involve lecturers from other programmes. The majority of SDPH offer programs according to a Bologna format, predominantly MPH, in addition to Bachelor and Doctoral studies. More than 80 masters are offered in the European Region, usually accredited at the national level. The highest number of teaching hours is still allocated to the classical subjects, ranking epidemiology highest with a median of 112 teaching hours followed by health systems and management with 100 hours (14). Emerging subjects like global health are presented in the curricula of 82% of SPDH; however, only 52% of them indicated the number of teaching hours with a medium of 40 hours, which nevertheless is a bit higher than recommended, e.g. for medical students as a minimum (30 hours) (52).

**Fig. 1:**
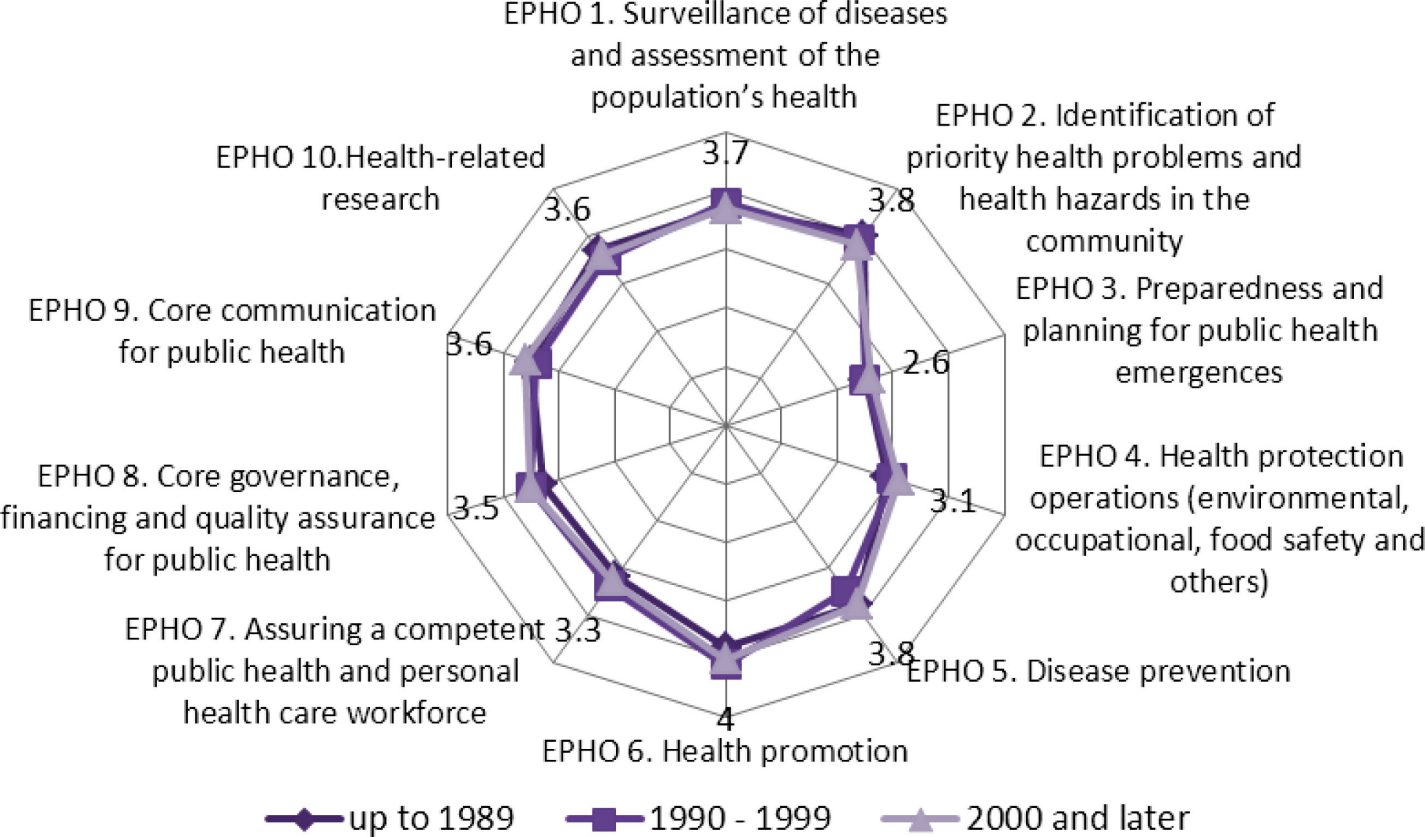
Outputs of Schools and Departments of Public Health in transferring knowledge and skills to assure the best possible performance of EPHOs and dynamic of establishment.^a^ ^a^ASPHER (54).

In general, the descriptive profile of SDPHs indicates significant diversity of institutions responsible for education and training in the field of public health in Europe. Relating the exit competences of graduates to the 10 Essential Public Health Operations (EPHOs) of WHO-EURO (53) shows that the capability of graduates to perform certain EPHOs after completion of a master programme is mediocre. Educational institutions in Europe assess their best output to be in the field of the EPHOs of health promotion, followed by disease prevention and identification of priority health problems and health hazards in the community. The least success they see is in the EPHOs dealing with preparedness and planning for public health emergencies, as a new emerging public health challenge. Though it was expected, it is obvious (see Fig. 1) that there is no difference in opinion about output in transferring knowledge and skills to graduates between schools and departments established at different times; and programmes are very much harmonised already.

**Fig. 2:**
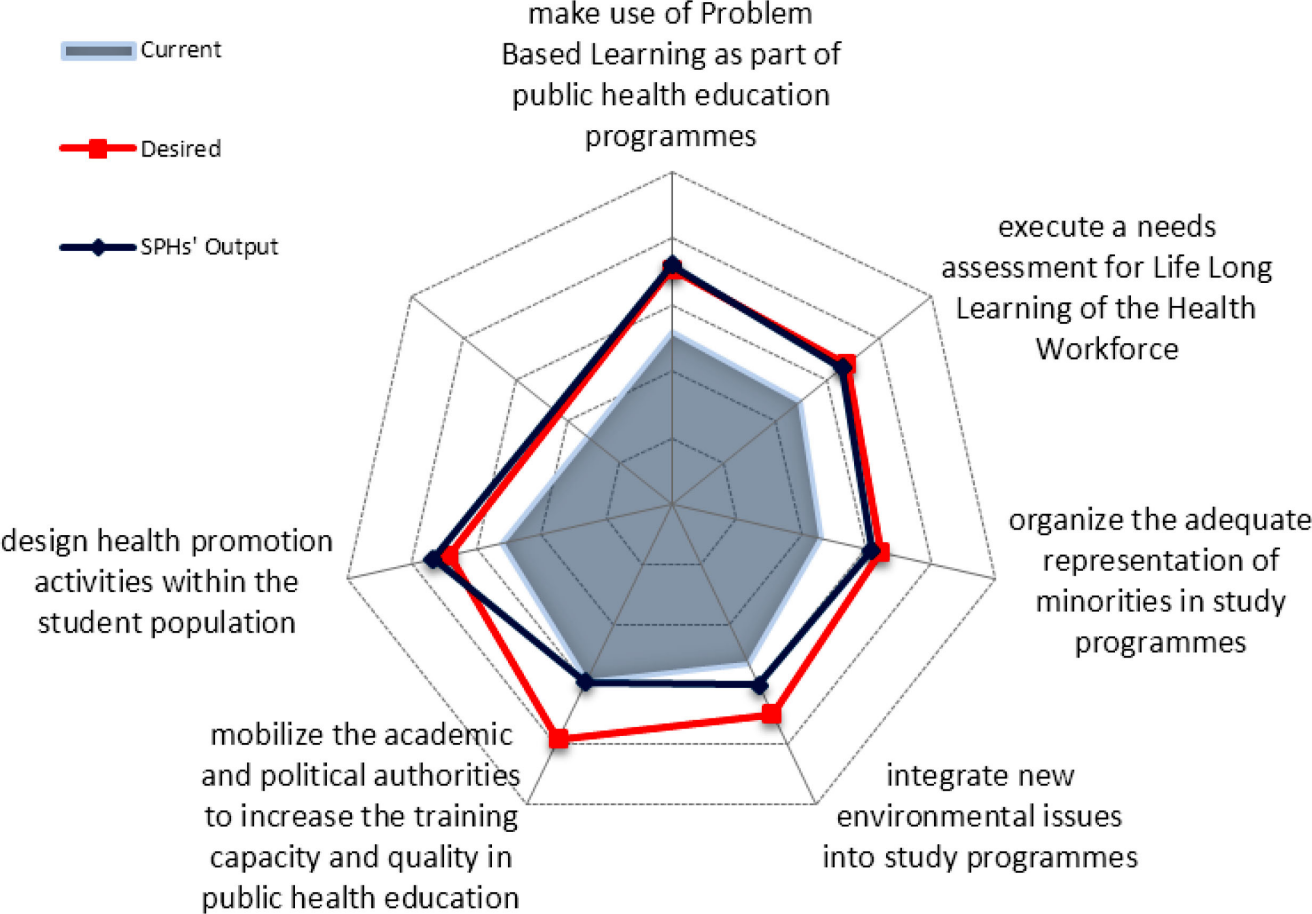
EPHO 7 on Assuring a competent public health and personal health care workforce.^a^ ^a^ASPHER (54).

Figure 2 presents detailed estimates of current and desired performance as regards single competences taking the example of EPHO 7 (assuring a competent public health and personal health care workforce). Sometimes gaps between current and desired levels as determined by the employers are less visible, but in general gaps are present within all competences across all EPHOs.

The desired performance by employers for most of the EPHOs (6/10) is almost congruent with the estimated output of SDPH. However, the current performance of employed public health professionals is considered to be lower than desired, leaving a gap between desired and current performance which may be due to older generations of professionals trained more than two decades ago. Therefore, European education for global health is in the forefront of rising needs for continuing education (CE) and professional development.

## Continuing professional development for global health based on online learning

Examples of good practice shared in the ASPHER survey (54) provide evidence that lifelong learning in multidisciplinary teams at the local and global level, based on critical reasoning to improve performance, is inspiring professionals for further professional development, including in global health. Innovative learning technologies (blended learning) provide incentives for public health professionals and academic staff to work together and to strengthen certain skills in the field of advocacy and negotiation for the benefit of health at the local level and global level. A major development in teaching during the last two decades is the use of online learning formats. In fact, e-Learning is ‘Information and Communication Technology Application to Learning’, and it can include all possible modalities: face-to-face learning in the traditional classroom setting, distance learning and any mix of the two called ‘Blended Learning’. But it can also be called ‘Computer-Supported Collaborative Learning’, ‘Technology Enhanced Learning’ or many other possible terms. The diffusion of technological advance and their adoption process has varied widely, barriers to change often being linked more to the resistance associated with human behaviour than to limitations of computer capabilities. However, the new generation of learners has an acceptance and utilisation of technology that can be much more advanced than that of their tutors.

The need for a better-trained workforce through lifelong learning and CE in the fields of public health is clearly recognised by public health professionals in Europe and worldwide. The SDPH survey pointed to the deficits still remaining in such efforts in the European Region as only less than one third of all respondents offer CE in terms of short courses, modules or summer schools. The duration and number of points according to the European Credit Transfer System/ECTS earned by participants during CE vary considerably, as well as the number of public health professionals enrolled. On average, the median per SDPH in Europe is 123, while the total number of participants in 2011 was as high as 6,209 professionals. In support of this is the fact that only a quarter of institutions indicate availability of modules for distance learning in order to support active and blended learning of students, on average seven of such modules per SDPH.

There are a number of reasons why blended learning is an appropriate mechanism for enhancing the quality of education in global health. First, students want to control their own learning. Students do not expect online courses to be easier. They do, however, expect the online learning environment to facilitate their success because they can review materials whenever they want and are more comfortable asking teachers for help. Online teachers see great benefits to student online learning, 76% (55) of educators believe that online learning benefits students by putting them in control of their own learning. Second, it is efficient. A comparison of outcomes of learning in face-to-face settings, through fully online learning and through blended learning showed that blended learning produced outcomes superior to either of the other conventional learning styles.

## Networking for global health education in Europe and beyond

European higher education institutions recognise the importance of partnerships both at regional and global level, and particularly in the field of global health. The whole Strategy for the External Dimension of the Bologna Process (26) strongly reflects the need for cooperation based on partnerships in a global setting and endorses ‘the principle that, in all matters related to higher education, academic values should prevail’. Harmonisation and standardisation of education in Europe (with recognition of professional qualifications) under the umbrella of the Bologna Process is opening a door for students’ and teachers’ mobility and professional exchange. There are various types of cooperation in higher education based on size and scope of institutional arrangements, with additional dimensions of integration, equity and intensity of cooperation, as recently is elaborated in the inspiring doctoral thesis: ‘Global Opportunities and Institutional Embeddedness. Higher Education Consortia in Europe and Southeast Asia’. In this work, Beerkens (56) highlighted, after extensive literature review: ‘An interesting paradox is that consortia, alliances or networks are based on compatibility as well as complementarities’. Complementarities refer predominantly to resources of institutional members of the network to reach defined objectives and are crucial for collaboration, while compatibility refers to the congruence of the background of partners. The paradox is coming from the importance of the diversity among members of a network, which should be controlled by leadership and management in order to achieve defined network goals.

To make a brief overview of networking for global health education, an extensive search of internet resources and organisational website reviews was conducted. The same positive paradox is observed in networking among academic and other institutions for global health (see Table 2). Some of networks are very active, which is visible at their internet presentations, while some others seem to be not so active. In Table 2, networks are presented according to regions defined by the World Health Organization.

**Table 2:** Selected networks involved in education and training for global health

Region	Network	Number of institutional members^a^
Africa	Association of Schools of Public Health in Africa (ASPHA) https://sites.google.com/site/asphaafricaorg/	26
	Global Health Workforce Alliance Members: Members and Partners in WHO African Region	93
Americas	American Association of Schools of Public Health (ASPH) Association of Schools and Programs of Public Health (ASPPH) (launches August 1, 2013) http://www.asph.org/	58 50+8
	Consortium of universities for global health (CUGH) http://www.cugh.org/about-us/mission	103
	The Latin American Alliance for Global Health (ALASAG) http://www.saludglobal.uchile.cl/english/index.php?option=com_content&view=article&id=126:the-latin-american-alliance-for-global-health-alasag-&catid=46:ourevents&Itemid=99	10
	Global Health Education Consortium (before 2005, under the name: Medical Health Education Consortium http://globalhealtheducation.org/SitePages/Home.aspx	90
	Latin American and Caribbean Association of Public Health Education (ALAESP) http://www.alaesp.sld.cu/	NA
	The American Society of Tropical Medicine and Hygiene (ASTMH) http://www.astmh.org/About_ASTMH.htm	
	Global Health Workforce Alliance Members: Members and Partners in WHO Region of the Americas http://www.who.int/workforcealliance/members_partners/amro/en/index.html	33
Asia – Pacific	Asia-Pacific Academic Consortium for Public Health (APACPH) http://www.apacph.org/wp/	83
	Global Health Workforce Alliance Members: Members and Partners in WHO South-East Asia Region http://www.who.int/workforcealliance/members_partners/searo/en/index.html	58
	Global Health Workforce Alliance Members: Members and Partners in WHO Western Pacific Region http://www.who.int/workforcealliance/members_partners/wpro/en/index.html	36
Eastern Mediterranean	Global Health Workforce Alliance Members: Members and Partners in WHO Eastern Mediterranean Region http://www.who.int/workforcealliance/members_partners/emro/en/index.html	26
	Public Health in the Arab World (PHAW) http://www.aub.edu.lb/fhs/phaw/Pages/index.aspx	34
Europe	Association of Schools of Public Health in the European Region (ASPHER) http://2011.aspher.org/	105 100+5
	European Academic Global Health Alliance (EAGHA) http://www.eagha.org/	36
	Global Health Europe http://www.globalhealtheurope.org/	30
	Federation of the European Societies for Tropical Medicine and International Health (FESTMIH) http://www.festmih.eu/Page/WebObjects/PageFestE.woa/wa/displayPage?name=Mission+Statutes	14
	tropED (Network for Education in International Health) http://www.troped.org/	20
	Medsin (The Medical Students International Network) http://www.medsin.org	30
	Federation of European Societies of Tropical Medicine and International Health (FESTMIH) http://www.festmih.eu/Page/WebObjects/PageFestE.woa/wa/displayPage?name=Home	15
	EUROLIFE Network of European Universities in Life Science http://eurolifeuniversities.org/	8
	Global Health Workforce Alliance Members: Members and Partners in WHO European Region http://www.who.int/workforcealliance/members_partners/euro/en/index.html	22
Global	Global Health Workforce Alliance Members: Academic and research institutions http://www.who.int/workforcealliance/members_partners/global/en/index.html	98
	World Federation of Public Health Associations (WFPHA) http://www.wfpha.org/about-us.html	79
	The International Federation of Medical Student Associations (IFMSA) http://www.ifmsa.org/About-Us	144
	International Union for Health Promotion and Education http://www.iuhpe.org/index.html?page=1&lang=en	16 15+1
	The Joint Action and Learning Initiative on National and Global Responsibilities for Health (JALI) http://www.jalihealth.org/	6

a One institution can belong to several networks.

They have different sizes, but quite similar scope of activities, and all of them have also activities related to education and training in public health and global health. Very often new networks are founded and later supported by well-established ones. A European example is the new network EAGHA (European Academic Global Health Alliance), established in 2010 and supported by ASPHER, which was founded in 1967. Networks for education in public health and global health predominantly have higher education institutions as members, but also governmental and non-governmental organisations involved in training. Besides sharing knowledge, experience and activities, networks for global health education are tending to overlap (one institution is belonging to several networks).

Some networks are intensively increasing the number of members. A prominent example is ASPHER, which increased its community by 30 new members in the last 5 years. The European approach to cooperation defined in the strategy as ‘European Higher Education in a Global Setting’ (26) is visible in this enlargement of ASPHER: five associated members come from outside of the European region, which is one of the objectives of the strategy: to establish cooperation beyond Europe.

Looking further, almost all networks have clear criteria for membership; however, some of them have divisions between full members, associate and affiliated members, based on research/educational capacity and presence of public health or global health academic programmes. It is also obvious that some networks are still searching to define SMART objectives (specific, measurable, achievable, relevant, and time-bounded objectives). This is visible in their actual predominant activities: during some of the time, education and training are dominant, and at other times research activities or public health interventions are. Regarding the scope of education for global health, it is an achievement that networks in public health are dealing with development of global health competences (‘Global Health is Public Health’), sometimes for varying groups of health professionals, e.g. medical doctors or medical students as pilot projects (see EAGHA).

Except for a few examples of good practice, there remains the challenge of planning, implementing, monitoring and evaluating of joint networks’ activities in the field of global health education and even more their potential impact on global population health in the future. Nevertheless, the significant achievement of these networks is visible (22) in the global arena of higher education, bringing enthusiasm (57), positive energy among members, very often expressed in their commitment to further global work and endorsement of strategic documents.

## Summary and conclusion

All relevant international organisations and academia agree on the central importance of a modernised teaching concept, based on core public health competences which should lead to the performance level requested by employers in the area of public health and global health. The Bologna Declaration, signed by almost all states, and similar agreements in other regions set the framework for Europe. Therefore, one could speak of a renaissance of public health. However, the process is slow and still incomplete, neither is there global agreement on the essential public health functions nor on the core competences for public health research and practice. Employers are involved hardly anywhere as a key reference point and practice links. A European accreditation agency has just started to operate. Furthermore the average European SDPH is too small to reach a critical mass for research and their total production of graduates is less than 1/3 of what is required following US estimates (14). During recent years, all actors, particularly the EU, have recognised the relevance of a concept of Lifelong Learning. Supported by blended or hybrid learning and employing online technology, these developments will change the educational landscape for all professionals and their networks and help make professionals more employable.

Finally, it should be recognised that for the public health workforce to truly be equipped to tackle current public health challenges, genuine leadership should exist at all levels. Leadership that is transformational and collaborative, not top-down, needs to be in place at the policy level, to bring about educational reform; at the teaching level, to implement change; and at the level of public health professionals, to put into practice the new skills.

As public health opportunities and threats are increasingly global, SDPH have to look beyond the national boundaries and participate in global networks for education, research and practice. ASPHER has started such development with EAGHA.

## Footnotes

^1^The target population comprised 80 full institutional members of the Association of Schools of Public Health in the European Region with a participation rate 82.5%. The web-based questionnaire covered institutional profiles and the ranking of exit competences for master of public health programmes, grouped according to WHO EPHOs (14).
